# Urban environmental noise and depression causal pathway: Potential role of chronic conditions as mediator

**DOI:** 10.1371/journal.pone.0322874

**Published:** 2025-05-07

**Authors:** Enrique Sanz Olea, Jesus León Hernández, Begoña Artíñano, Verónica Briz, Spyros Karakitsios, Dimosthenis Sarigiannis, Saul García Dos Santos, Beatriz Nuñez-Corcuera, Rebeca Ramis

**Affiliations:** 1 Hospital Universitario La Paz, Madrid, Spain; 2 Hospital General Universitario de Ciudad Real, Ciudad Real, Spain; 3 Environment Department, CIEMAT, Madrid, Spain; 4 Viral Hepatitis Reference and Research Laboratory, National Center of Microbiology, Institute of Health Carlos III, Madrid, Spain; 5 HERACLES Research Center – KEDEK, Aristotle University of Thessaloniki, Thessaloniki, Greece; 6 Department of Chemical Engineering, Environmental Engineering Laboratory, Aristotle University of Thessaloniki, Thessaloniki, Greece; 7 Environmental Health Engineering, School for Advanced Study IUSS, Pavia, Italy; 8 Area de Contaminación Atmosférica, Centro Nacional de Sanidad Ambiental, Instituto de Salud Carlos III, Madrid, Spain; 9 Cancer and Environmental Epidemiology Unit, National Centre for Epidemiology, Instituto de Salud Carlos III, Madrid, Spain; 10 Spanish Consortium for Research on Epidemiology and Public Health (CIBERESP), Instituto de Salud Carlos III, Madrid, Spain; Universitat de Barcelona, SPAIN

## Abstract

**Background:**

Accepting the fact of a growing urban population and associated health risks, such as increased exposure to pollutants, and considering the high prevalence of chronic diseases, this study aims to investigate the relationship between exposure to environmental noise and depression. The primary objective is to determine the potential mediating role of chronic diseases in this relationship. This study is part of the European H2020 URBANOME project, designed to explore the relationship between the environment and health in urban settings. The main goal of the project is to promote urban health, well-being, and liveability by systematically integrating health concerns into urban policies and civic activities.

**Materials and methods:**

We obtained the data for this study from the Madrid City Health Survey, conducted through computer-assisted telephone interviews. Outcome variables assessed through self-reports included depression, exposure to environmental noise, and the presence of chronic diseases. We used a counterfactual mediation framework, implemented by the R package multimediate, to evaluate the potential mediating role of chronic diseases in the relationship between exposure to environmental noise and depression.

**Results:**

The study included 8,445 interviews, with a higher percentage of women (54.67%) than men. 23.29% were over 65, and 17.17% under 30. Notably, 7.82% reported depression, 39.53% had chronic diseases, and 35.43% acknowledged noise exposure. In our regression models, those exposed to environmental noise were 1.24 times more likely to have a chronic disease, and individuals with a chronic disease were 2.93 times more likely to suffer from depression. Participants exposed to environmental noise had 1.03 times more depression, being the 13% of the noise health effect mediated by the presence of chronic illness.

**Conclusion:**

Our findings suggest a link between environmental noise exposure and depression, potentially mediated by chronic diseases. This points out the need for public health interventions to reduce urban noise exposure and improve mental health. Furthermore, prospective studies are needed for confirmation, incorporating noise level measurements and temporal data on the onset of chronic diseases and depression.

## Introduction

According to United Nations the urban population worldwide for 2021 is 56% of the global total and to by 2050 it will be 68% [1]. This makes the urban environment an important determinant of human health and wellbeing.

Environmental urban stressors include, among others, noise (aircraft, housing, industries, traffic and railway) and atmospheric pollutants (PM2,5 particulate matter, NO2, O3, etc,), Clearly the intensity of these are close related to high emission sources, high-density housing and traffic, inadequate public transportation, improper road and urban area design and maintenance, and a lack of green spaces [2]. There is substantial evidence regarding the impact of urban stressors on population health. Urban exposure to noise and air pollution is known to increase the likelihood of specific chronic diseases such as hypertension, cardiovascular problems, sleep disorders, and depression and loss of life years [3–6]. The complex emerging issues generated by urban stressors pose a challenge to sustainable development, well-being, and the quality of life in urban populations [2].

Noise can be defined as unwanted sound. Environmental noise encompasses all unwanted noise in our community [7]. With the growth of cities, noise sources have increased in number and intensity, having a much greater impact on public health [8,9]. Despite this increase in noise sources, traffic noise remains the primary health stressor [6,10]. Traffic noise is an environmental risk factor for various diseases, including hearing loss, sleep disturbances, mental health issues, and cardiovascular diseases [7]. A report from the World Health Organization (WHO) estimates that at least one million disability-adjusted life years (DALYs) are lost each year due to diseases related to traffic noise in Western Europe [6].

Depression is highly prevalent, with a lifetime prevalence rate of 20.6% in the United States [11], and around 6.4%,in Europe, although there is considerable variability among countries [12]. It is estimated to affect 280 million people worldwide, with 5% of adults and 5.7% of adults over 60 years of age experiencing it [13]. Depression is one of the most common mental disorders and a leading cause of disability worldwide [14]. There are numerous risk factors associated with depression, and as of today, a comprehensive understanding of them and the multiple interactions among these factors have not been fully achieved [15]. Among the most common risk factors related to depression are physical pain resulting from certain illnesses, chronic diseases, personal and familial history of depression, adverse life events such as job loss, loneliness, family problems, insomnia, and sleep disturbances, among others [16]. Concerning insomnia, research has demonstrated that noise induces various stress reactions and insomnia. These factors, in conjunction with chronic noise exposure, may predispose individuals to depression [17,18].

Cardiovascular diseases are the leading cause of morbidity and mortality worldwide despite numerous advances in prevention and treatment [19]. There is evidence that environmental stress factors play a role in the development of cardiovascular diseases. Various epidemiological studies have shown an association between environmental noise exposure, especially from traffic sources, and an increased risk of cardiovascular diseases [9,20–22]. Therefore, traffic noise is considered a cardiovascular risk factor [23]. Combined findings from observational and experimental studies have provided insights into the underlying mechanisms of noise-induced vascular dysfunction and cardiovascular diseases. Although not fully elucidated, it is suggested that noise can cause disruptions in sleep structure, increases in stress hormone levels, oxidative stress, endothelial dysfunction, and high blood pressure, ultimately leading to an increased risk of cardiovascular diseases [3–5].

Chronic diseases and depression have a bidirectional relationship, meaning the onset of one predisposes to the development of the other [24]. There is evidence that both patients with depression are more likely to suffer from chronic diseases [25], and that patients with chronic diseases are more prone to depression [26]. In the case of cardiovascular diseases and depression, it is believed that risk factors act as mediators [27,28]. Depression would have a negative impact on diet, exercise, substance consumption, as well as medical treatment adherence, leading to exacerbation of cardiovascular diseases in those already affected and predisposing them in healthy individuals [29]. Additionally, patients diagnosed with a chronic disease, upon realizing the chronic nature of their condition or when complications arise, may develop mental disorders such as depressive episodes [24]. From a pathophysiological perspective, depression induces an increase in inflammatory activity and a state of hypercoagulability that may eventually lead to cardiovascular diseases [30].

As it mentioned above, noise has become an important environmental stressor, mainly in cities, and the study of its direct and indirect effect on health is an emerging need. Accordingly, the URBANOME project [31] was set up to investigate the relationships between the environment and health in urban settings. The project main goal is to promote urban health, well-being, and liveability by systematically integrating health concerns into urban policies and citizen activities. Under this umbrella, with this study, we aim to study the role of chronic conditions as a mediator in the casual pathway linking urban environmental noise to depression. To our knowledge not other study has been set up to evaluate this potential mediation link.

## Materials and methods

Within the URBANOME project, the Madrid city is considered an urban living lab to address citizens health concerns regarding ageing, physical and mental health and their relationship with environmental stressors and social equality. The city of Madrid is the capital of Spain and the most populated city in the country. Up to 1st January 2024, the municipality area has 3,339,931 inhabitants [32]. The 66.2% of these are aged between 16 and 64 years. The city is divided in 21 districts. The most populated district is in the southwest area Carabanchel (total habitants 262.339, 18 674 hab/km^2^) followed by Fuencarral-El Pardo in the northwest area (total habitants 248.443, 1044.59 hab./km²). The overall numbers show that only five districts have more than 200,000 inhabitants while three has less than 100.000. Five of them are between 150.0000 and 200,000 and most districts are ranged between 100,000 and 150,000 inhabitants. In terms of social inequality, the city data from 2021 shows a clear socioeconomic gradient north-south when comparing the average income [33].

The study design was a cross-sectional study with the data from the City of Madrid Health Survey conducted by the public organization “Madrid Salud” [34]. This survey is carried out every four years using a stratified random sampling method that includes individuals aged 15 or older residing in the city of Madrid. Data collection was done through computer-assisted telephone interviews (CATI), with a total of 8,445 interviews conducted. The interviews took place from October 9th to December 10th, 2,017. The survey was directed at the Madrid population and investigated various aspects, including self-reported health, quality of life related to health, the presence of chronic diseases and disabilities, exposure to environmental noise, symptoms of depression, substance and gambling addictions, and other factors related to lifestyles, the healthcare system, and social determinants, with a particular focus on living and working conditions.

The studied variables, depression, exposure to environmental noise and presence of chronic disease were assessed through self-reporting by the interviewee. Depression status was determined based on those who answered “yes” to the following statement, “I am going to read you a list of diseases or health problems (depression), for each one, tell me if your doctor has told you that you have it or not.” Exposure to environmental noise was evaluated based on those who responded “yes” to the following statemen, “Are you bothered by environmental noises heard from your home?” The presence of chronic disease was assessed with those who answered “yes” to the following statement, “Do you have any chronic or long-term health problems?” Moreover, it was considered a long-term issue if the health problem or disease had lasted or was expected to last for 6 months or more.

Data were collected on the respondents’ gender, age, categorized as individuals between 15 and 29, 30 and 44, 45 and 64, and finally over 65 years old. Similarly, variables related to risk factors such as sleep quality, smoking habits, and body mass index (BMI), the last was classified as less than 18.50 kg/m^2^, between 18.50 and 25 kg/m^2^, between 25 and 30 kg/m^2^, and over 30 kg/m^2^. Unfortunately, the variable sleep quality was not complete for half of the participants. Finally, data were compiled on the district of residence within the City of Madrid at the time of the survey.

Lastly, we included data on social vulnerability at district level. We took this information from the district vulnerability index developed by Madrid City Council [35]. This annual vulnerability ranking of districts and neighborhoods was put together with the purpose of better understanding the situation of each Madrid district and the relationship among social vulnerability, environmental factors, and health status. So, this information can be used as a basis for public policy design and to combat risks of both exclusion and emergency of the citizens living inside of each particularly vulnerable area. The district vulnerability index is given by a number that indicates its degree of vulnerability for each district. So, the higher the number, the greater the vulnerability would be. It is calculated based on the average of population data (immigration rate, life expectancy, either no education or primary education), socio-economic status (average household income), economic activity (absolute unemployment rate, unemployment rate for those over 45, unemployment rate without benefits), urban development (cadastral value), and welfare needs (rate of dependent care demand, families receiving minimum income, rate of tele-assistance for dependency).

### Conceptual framework of mediation analysis

**Fig 1:**
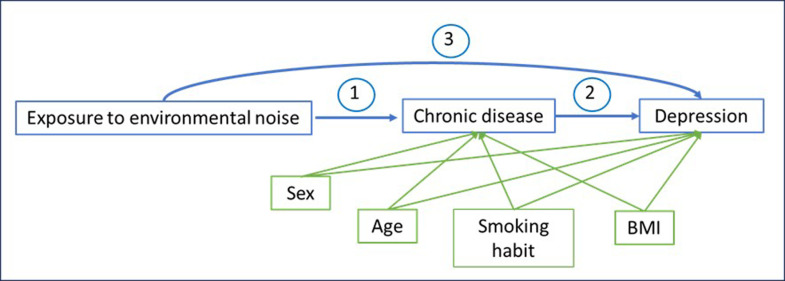
Conceptual mediation model with covariates.

The aim of causal mediation analysis is to find out how much of the total effect following a well-defined exposure is in turn partly due to an intermediate variable involved in the causal pathway (indirect effect). In our mediation framework, we assessed whether the effect of exposure to environmental noise on depression cases could be explained (i.e., mediated) by the presence of chronic disease that was also link to environmental noise exposure. Our conceptual model can be easily presented in the below DAG (Directed Acyclic Graph) shown in Fig 1. An example with an specific outcome would be that exposure to environmental noise could lead to cardiovascular disease -through arterial hypertension, endothelial dysfunction, oxidative stress, and inflammation, as in pointed out in recent research [4,36–38] (arrow 1). And chronic cardiovascular disease, triggered by the aforementioned mechanisms due to noise, may contribute to lower mood states and impair individuals in their daily tasks, potentially leading to depression [24,39,40] (arrow 2). Additionally, exposure to environmental noise has also been directly associated with depression, mainly through sleep disturbances [17,18] (arrow 3).

In our analysis, we formally tested this possible mediating role of chronic disease as an intermediary variable in the association between exposure to environmental noise and depression. We used the counterfactual mediation framework to estimate the natural indirect (i.e., mediated) effects, that is the standard approach to mediation analysis, implemented the models by the mediation R package [41]. In this setting, our mediator models were logistic models where depression entered as the dependent variable in separate models (2 and 3 arrows in Fig 1) and exposure to environmental noise was entered as the independent variable (1 and 3 arrows in Fig 1).

The logistic regression models and the mediation model included the variables of exposure to environmental noise, presence of chronic illness, and depression. Both outcome and mediator models were adjusted for covariates such as age, sex, BMI, smoking status and district.

### Mediation analysis models

- M1: Chronic disease ~ Noise + Covariates
- M2: Depression ~ Chronic disease + Covariates
- M3: Depression ~ Noise + Covariates
- Full Model (FM): Depression ~ Noise + Chronic disease + Covariates.

As a result, absolute mediated effects (natural indirect effects) were reported as the increase in cases of depression related to exposure to environmental noise attributed to the presence of chronic disease. The relative mediated effect was calculated as the ratio between the mediated effects and the adjusted changes in depression cases, depending on whether they were exposed to environmental noise before adding the presence of chronic disease to the model.

## Results

The study included 8,445 interviews. Table 1 shows the sample description values of percentage and the total frequencies of the relevant variables for our study. In summary, more women (54.67%) were interviewed than men. 23.29% were over 65 years old, and 17.17% were under 30 years old. Regarding the study main variables, 7.82% reported having depression, 39.53% had some chronic disease, and 35.43% acknowledged being exposed to environmental noise.

**Table 1:** Sample description values such as the percentage along with the frequencies of responses.

Main variables	Percentage	Total
**Depression**	**7.82%**	**692**
**Chronic disease**	**39.53%**	**3496**
**Exposure to environmental noise**	**35.42%**	**3133**
***Sex***		
Male	45.33%	4009
Female	54.67%	4836
***Age***		
15-29 years	17.17%	1519
30-44 years	27.07%	2394
45-64 years	32.47%	2872
>65 years	23.29%	2060
***Sleep quality***		
Very good	9.37%	829
Good	24.70%	2185
Regular	13.28%	1175
Bad	1.92%	170
Very bad	0.67%	59
NA	50.05%	4427
***Smoking habit***		
Daily smoker	19.04%	1684
Non-daily smoker	2.65%	234
Former smoker	30.19%	2670
Never smoker	48.11%	4255
NA	0.02%	2
***BMI***		
<18,50	2.60%	230
18,50-25	50.75%	4489
25-30	33.25%	2941
>30	11.26%	996
NA	2.14%	189
***Madrid district name***		
Arganzuela	4.73%	418
Barajas	4.74%	419
Carabanchel	4.85%	429
Centro	4.70%	416
Chamartín	4.69%	415
Chamberi	4.74%	419
Ciudad Lineal	4.90%	433
FuencarralEl Pardo	4.79%	424
Hortaleza	4.87%	431
Latina	4.86%	430
MoncloaAravaca	4.82%	426
Moratalaz	4.67%	413
Puente de Vallecas	4.93%	436
Retiro	4.74%	419
Salamanca	4.69%	415
San Blas/Canillejas	4.74%	419
Tetuan	4.67%	413
Usera	4.75%	420
Vicalvaro	4.74%	419
Villa de Vallecas	4.66%	412
Villaverde	4.74%	419

NA, no answer.

**Fig 2:**
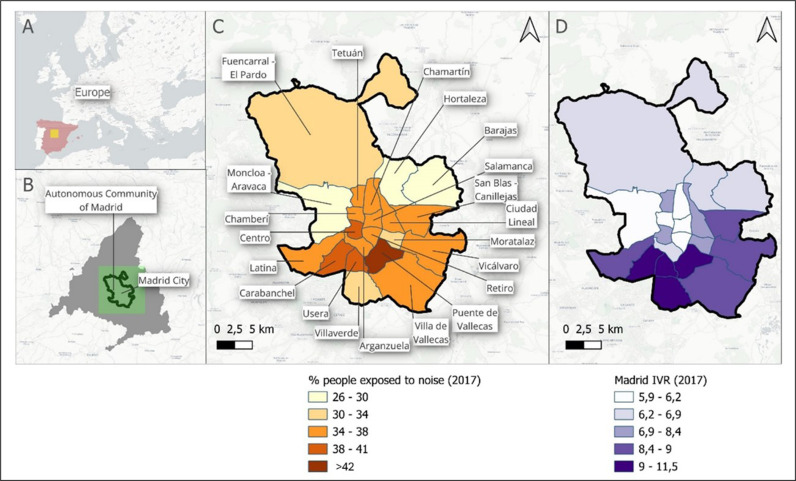
A: Europe map. B: Central Spain map. C: The % of reported noise exposure by districts map. D: Vulnerability index map.

Fig 2 shows the spatial district distribution of the exposure to noise is showed. Maps A and B, show the location of Madrid City within Europe and Spain. Maps C and D present the city of Madrid, subdivided into its districts, where Map C displays the percentage of reported noise exposure (the individuals who answered yes to being exposed to environmental noise out of the total number of respondents of the City of Madrid Health Survey conducted by the public organization “Madrid Salud” [34]) and Map D presents the vulnerability index. Looking at Map C it is evident that districts in the southern region, such as Puente de Vallecas and Carabanchel, experience higher noise exposure, whereas northern districts like Moncloa-Aravaca are less affected. When comparing with Map D, generally south and south-east districts are those that exhibit greater vulnerability, and again the Puente de Vallecas presents the higher value (11.5).

Also, a correlation analysis was conducted to investigate the potential relationship between vulnerability and exposure to environmental noise. Although the correlation was low (0.331), it was noted that the district with the highest vulnerability, Puente de Vallecas, is also the most exposed to environmental noise. Similarly, it was observed that southern districts like Usera and Carabanchel have high levels of both vulnerability and noise exposure. Conversely, northern districts such as Fuencarral-El Pardo, Barajas, Hortaleza, and Moncloa-Aravaca exhibit low levels of both environmental noise exposure and vulnerability (Fig 2).

Table 2 shows the odds ratios (OR) and their respective 95% confidence intervals (CI 95%) for the study covariates included in the model. The results indicated a higher risk of depression among those exposed to environmental noise and those suffering from chronic diseases. Table 2 shows that those exposed to environmental noise had a 1.47 times higher risk of depression, with a 95% confidence interval ranging from 1.24 to 1.74 and a p-value <0.001. Similarly, individuals with chronic diseases had a 2.88 times higher risk of experiencing depression, with a 95% CI ranging from 2.42 to 3.44 and a p-value <0.001.

**Table 2:** Odds ratios (OR) and their respective 95% confidence intervals (CI 95%) for depression in the full model.

Covariates	Total exposed (8445 total) (% exposed)	OR	95% CI	p-value
(Intercept)		0.01	(0.01,0.02)	<0.001
**Exposure to environmental noise**	**3133 (37%)**	**1.47**	**(1.25,1.74)**	**<0.001**
**Chronic disease**	**3496 (38%)**	**3.03**	**(2.55,3.61)**	**<0.001**
Sex male*	**4009 (35%)**		**–**	**–**
**Sex female**	**4836 (36%)**	**2.77**	**(2.29,3.24)**	**<0.001**
15-29 years*	**1519 (33%)**		**–**	**–**
**30-44 years**	**2394 (36%)**	**1.74**	**(1.24,2.45)**	**<0.001**
**45-64 years**	**2872 (40%)**	**2.36**	**(1.70,3.27)**	**<0.001**
**>65 years**	**2060 (29%)**	**2.74**	**(1.95,3.84)**	**<0.001**
Former smoker*	2670 (37%)		–	–
Never smoker	4255 (33%)	1.19	(0.98,1.45)	0.8
**Daily smoker**	**1684 (37%)**	**1.72**	**(1.36,2.16)**	**<0.001**
Non-daily smoker	234 (38%)	1.17	(0.64,2.13)	0.61
IMC < 18,50*	230 (36%)		–	–
IMC 18.50–25	4489 (36%)	0.69	(0.43,1.12)	0.13
IMC 25–30	2941 (34%)	0.81	(0.49,1.32)	0.39
IMC > 30	996 (36%)	1.12	(0.67,1.87)	0.66
IMC NA	189 (30%)	0.50	(0.24,1.04)	0.06
Arganzuela District	418 (38%)		–	–
Barajas District *	419 (26%)	1.34	(0.78,2.28)	0.29
Carabanchel District	429 (40%)	1.27	(0.76,2.14)	0.36
**Centro District**	**416 (38%)**	**1.92**	**(1.16,3.19)**	**0.01**
Chamartín District	415 (36%)	0.99	(0.57, 1.74)	0.98
Chamberí District	419 (38%)	0.94	(0.53, 1.64)	0.82
Ciudad Lineal District	433 (35%)	1.24	(0.74, 2.09)	0.42
Fuencarral El Pardo District	424 (33%)	0.72	(0.39, 1.30)	0.28
Hortaleza District	431 (25%)	1.35	(0.80, 2.28)	0.26
Latina District	430 (36%)	1.14	(0.67,1.92)	0.63
Moncloa-Aravaca District	426 (29%)	0.96	(0.55,1.69)	0.89
Moratalaz District	413 (32%)	0.83	(0.47,1.46)	0.52
Puente de Vallecas District	436 (46%)	1.29	(0.77,2.15)	0.33
Retiro District	419 (35%)	0.94	(0.54,1.63)	0.83
Salamanca District	415 (35%)	0.89	(0.51,1.56)	0.68
San Blas-Canillejas District	419 (34%)	1.48	(0.89,2.46)	0.13
Tetuán District	413 (38%)	1.15	(0.68,1.96)	0.60
Usera District	420 (40%)	1.20	(0.71,2.02)	0.49
Vicálvaro District	419 (35%)	1.12	(0.65,1.93)	0.67
**Villa de Vallecas District**	**412 (37%)**	**1.91**	**(1.16,3.14)**	**0.01**
Villaverde District	419 (34%)	1.56	(0.94,2.58)	0.08

Table 3 presents the odds ratios (OR) and their respective 95% confidence intervals (95% CI) for the covariates from the logistic regression analysis between exposure to environmental noise and chronic diseases. Individuals exposed to environmental noise had a 1.24 times higher risk of chronic disease, with a 95% confidence interval ranging from 1.13 to 1.37 and a p-value <0.001, without including the presence of depression.

**Table 3:** Odds ratios (OR) and their respective 95% confidence intervals (95% CI) for chronic disease on M1: Chronic disease ~ Noise + Covariates.

Covariates	Total exposed (8445 total)	OR	95% CI	p-value
(Intercept)		0.35	(0.24,0.51)	<0.001
**Exposure to environmental noise**	**3133 (37%)**	**1.27**	**(1.16,1.40)**	**<0.001**
Sex male*	4009 (35%)	1.00	–	–
**Sex female**	**4836 (36%)**	**1.29**	**(1.17,1.41)**	**<0.001**
15-29 years*	1519 (33%)	1.00	–	–
**30-44 years**	**2394 (36%)**	**1.23**	**(1.05,1.43)**	**0.01**
**45-64 years**	**2872 (40%)**	**2.21**	**(1.91,2.56)**	**<0.001**
**>65 years**	**2060 (29%)**	**3.63**	**(3.10,4.24)**	**<0.001**
Former smoker*	2670 (37%)	1.00	–	–
**Never smoker**	**4255 (33%)**	**0.76**	**(0.68,0.84)**	**<0.001**
**Daily smoker**	**1684 (37%)**	**0.83**	**(0.72,0.95)**	**0.01**
Non-daily smoker	234 (38%)	0.81	(0.60,1.09)	0.17
IMC < 18,50*	230 (36%)	1.00	–	–
IMC 18.50–25	4489 (36%)	0.93	(0.69,1.25)	0.61
IMC 25–30	2941 (34%)	1.18	(0.87,1.59)	0.28
**IMC > 30**	996 (36%)	**2.07**	**(1.50,2.84)**	**<0.001**
IMC NA	189 (30%)	1.16	(0.76,1.75)	0.49
District Arganzuela*	418 (38%)	1.00	–	–
**District Barajas**	**419 (26%)**	**0.75**	**(0.56,0.99)**	**0.05**
District Carabanchel	429 (40%)	0.84	(0.63,1.12)	0.22
**District Centro**	**416 (38%)**	**0.73**	**(0.55,0.98)**	**0.04**
**District Chamartín**	415 (36%)	0.71	(0.53,0.95)	0.02
District Chamberi	419 (38%)	0.80	(0.60,1.07)	0.14
District Ciudad Lineal	433 (35%)	0.84	(0.63,1.11)	0.22
District Fuencarral El Pardo	424 (33%)	0.76	(0.56,1.01)	0.06
District Hortaleza	431 (25%)	0.89	(0.67,1.19)	0.43
District Latina	430 (36%)	0.95	(0.70,1.24)	0.70
**District Moncloa-Aravaca**	**426 (29%)**	**0.74**	**(0.55,0.99)**	**0.04**
District Moratalaz	413 (32%)	0.87	(0.65,1.16)	0.34
District Puente de Vallecas	436 (46%)	0.87	(0.65,1.16)	0.33
District Retiro	419 (35%)	0.80	(0.60,1.07)	0.13
District Salamanca	415 (35%)	0.88	(0.66,1.17)	0.38
District San Blas-Canillejas	419 (34%)	0.89	(0.65,1.18)	0.41
District Tetuan	413 (38%)	0.96	(0.72,1.27)	0.76
District Usera	420 (40%)	1.02	(0.75,1.36)	0.89
**District Vicalvaro**	**419 (35%)**	**0.69**	**(0.52,0.93)**	**0.01**
District Villa de Vallecas	412 (37%)	0.88	(0.66,1.17)	0.38
District Villaverde	419 (34%)	0.98	(0.74,1.31)	0.92

*Reference category for the categorical covariates.

Table 4 displays the odds ratios (OR) and their respective 95% confidence intervals (CI 95%) for the covariates from the logistic regression analysis between chronic diseases and depression, excluding exposure to environmental noise. Individuals with chronic diseases had a 2.93 times higher risk of depression, with a 95% confidence interval ranging from 2.46 to 3.50 and a p-value <0.001, without including exposure to environmental noise.

**Table 4:** Odds ratios (OR) and their respective 95% confidence intervals (95% CI) for depression on M2: Depression ~ Chronic disease + Covariates.

Covariates	Total exposed (8445 total)	OR	95% CI	p-value
(Intercept)		0.01	(0.01, 0.02)	<0.001
**Chronic disease**	**3496 (38%)**	**3.10**	**(2.60, 3.69)**	**<0.001**
Sex male*	4009 (35%)	1.00	–	–
**Sex female**	**4836 (36%)**	**2.79**	**(2.31, 3.36)**	**<0.001**
15–29 years*	1519 (33%)	1.00	–	–
30–44 years	**2394 (36%)**	1.76	(1.25, 2.47)	0
**45-64 years**	**2872 (40%)**	**2.42**	**(1.75, 3.36)**	**<0.001**
**>65 years**	**2060 (29%)**	**2.67**	**(1.91, 3.74)**	**<0.001**
Former smoker*	2670 (37%)	1.00	–	–
Never smoker	**4255 (33%)**	1.11	(0.97,1.44)	0.1
**Daily smoker**	**1684 (37%)**	**1.70**	**(1.35, 2.14)**	**<0.001**
Non-daily smoker	234 (38%)	1.17	(0.64, 2.14)	0.57
IMC <18.50*	230 (36%)	1.00	–	–
IMC 18.50–25	4489 (36%)	0.69	(0.43, 1.10)	0.16
IMC 25–30	2941 (34%)	0.79	(0.49, 1.29)	0.41
IMC >30	996 (36%)	1.10	(0.66, 1.87)	0.55
IMC NA	189 (30%)	0.49	(0.23, 1.01)	0.07
District Arganzuela*	418 (38%)	1.00	–	–
District Barajas	**419 (26%)**	1.26	(0.74, 2.15)	0.37
District Carabanchel	429 (40%)	1.30	(0.77, 2.18)	0.3
**District Centro**	**416 (38%)**	**1.93**	**(1.16, 3.20)**	**0.01**
District Chamartín	415 (36%)	0.97	(0.56, 1.70)	0.95
District Chamberi	419 (38%)	0.92	(0.53, 1.61)	0.78
District Ciudad Lineal	433 (35%)	1.22	(0.73, 2.06)	0.57
District Fuencarral El Pardo	424 (33%)	0.70	(0.38, 1.25)	0.22
District Hortaleza	431 (25%)	1.27	(0.75, 2.14)	0.37
District Latina	430 (36%)	1.12	(0.66, 1.89)	0.73
District Moncloa-Aravaca	**426 (29%)**	0.91	(0.51, 1.61)	0.71
District Moratalaz	413 (32%)	0.82	(0.45, 1.45)	0.43
District Puente de Vallecas	436 (46%)	1.34	(0.81, 2.23)	0.31
District Retiro	419 (35%)	0.93	(0.50, 1.53)	0.67
District Salamanca	415 (35%)	0.88	(0.50, 1.53)	0.63
District San Blas-Canillejas	419 (34%)	1.45	(0.87, 2.42)	0.18
District Tetuan	413 (38%)	1.16	(0.68, 1.96)	0.58
District Usera	420 (40%)	1.22	(0.72, 2.05)	0.58
District Vicalvaro	**419 (35%)**	1.12	(0.65, 1.93)	0.76
**District Villa de Vallecas**	412 (37%)	**1.90**	**(1.15, 3.13)**	**0.02**
District Villaverde	419 (34%)	1.53	(0.93, 2.53)	0.16

*Reference category for the categorical covariates.

Table 5 presents the results of the mediation analysis. Control group were those individuals that did not report environmental noise, and the treated group were those who did reported it. Here it was assessed that in the relationship between exposure to environmental noise and the presence of depression, the presence of chronic diseases could act as a mediator. In the relationship between exposure to environmental noise and the presence of depression, the proportion mediated (Prop. Mediated) by chronic diseases would be 13% (Prop.Mediated), with a confidence interval ranging from 7% to 24%, and it is statistically significant with a p-value <0.001. This proportion represents the average between the proportion mediated by chronic diseases in those not exposed to environmental noise 11% (Prop.Mediated (control)) and the proportion mediated in those who are exposed to environmental noise 15% (Prop.Mediated (treated)).

**Table 5:** Estimate, odds ratios (OR) and their respective 95% confidence intervals (95% CI) for the mediation analysis.

	Estimate	95% CI	p-value	OR	95% CI	p-value
Total effect	0.03	(0.02, 0.04)	<2e-16	1.03	(1.02, 1.04)	<2e-16
Prop. Mediated (control)	11%	(5%, 22%)	<2e-16			
Prop. Mediated (treated)	15%	(8%, 26%)	<2e-16			
ACME (average)	0.004	(0.00, 0.01)	<2e-16	1.004	(1.00, 1.01)	<2e-16
ADE (average)	0.03	(0.02, 0.04)	<2e-16	1.03	(1.02, 1.04)	<2e-16
**Prop. Mediated**	**13%**	**(7%, 24%)**	**<2e-16**			

*ACME: average causal mediaton effects. ADE: average direct effects. Prop. Mediated: proportion mediated.

In summary, the results for all the analysis showed that participants that reported environmental noise were 1.24 times more likely to have chronic illness compared to those that did not report; that participants with chronic illness were 2.93 times more likely to experience depression compared to those without chronic illness; and participants exposed to environmental noise had 1.03 times more depression, being the 13% of the noise health effect mediated by the presence of chronic illness.

## Discussion

In this study, the effects of exposure to environmental noise on depression cases were investigated in the City of Madrid. From the beginning the hypothesis was that this relationship could be mediated by the presence of chronic illness, therefore, a mediation analysis was conducted. The findings supported that the presence of chronic illness could act as a mediator in the relationship between exposure to environmental noise and depression.

In this study, the environmental noise exposure variable was self-reported by the survey respondents. Other studies have also employed different approaches. The majority of studies utilized models to predict noise levels and then assign them to residential location, such as measuring noise exposure using historical monitoring data, traffic exposure estimates, and digital landscape models [42–44]. A different approach was used in France by Nassur et al. [5]. to assess noise exposure during sleep, sensors were deployed within the participants’ residences. Subsequently, algorithms were employed to analyse the collected data and calculate noise exposure levels within the household. As we mention above, our method was self-reported, but we believe that this approach to environmental noise exposure has certain advantages in comparison to the models. Firstly, by focusing on self-perceived noise, we can better understand the patient’s own experience, as noise, psychologically speaking, does not affect all individuals equally. Considering that our outcome variable is depression, knowing the patient’s subjective experience is of great importance. Secondly, there are various circumstances beyond the individual’s experience that condition exposure to environmental noise, such as living far from traffic jams or urban centers, as well as the important variable of house noise insulation. By understanding the patient’s personal experience, we are indirectly taking these factors into account. Despite all the possible circumstances that influence noise, the main issue is each person either if he hears environmental noise or is exposed to it.

With reference to noise and depression the first study that directly assessed and compared the risks of depression associated with aircraft, traffic, and railway noise was conducted in 2017 by Andreas Seidler et al. [42]. They found higher risk of depression among those exposed to noise from all sources, whether it was aircraft, traffic, or railway noise, with an even higher risk if the noise sources were combined. This aligns with our findings, where we also observed an increased risk of depression among those exposed to environmental noise. However, the studies are not directly comparable because we assessed noise exposure differently; we did not categorize exposure by different noise sources and did not include data on exposure levels in decibel (dB). In the Finnish study published in 2014 by Halonen Jl et al. [43], no association was found between noise exposure and the use of psychotropic medication. However, they did find an association between exposure to environmental noise and a poorer self-perceived of health. These discrepancies with our study’s findings may be attributed to several factors. Firstly, they utilized a geolocation model to estimate noise exposure within the household, which differs from our self-reported noise exposure approach. Secondly, many people with depression are not diagnosed and therefore do not take any kind of medication, additionally, the term “psychotropic medication” encompasses a range of medications beyond just antidepressants, potentially introducing confounding factors. Their comparison of noise levels <45 dB with >60 dB might have oversimplified the exposure categories. Moreover, socio-cultural characteristics and behaviour of the Finnish population and their building typology. In fact, the widespread use of double-glazed windows due to the long cold weather period, could have influenced the results. These differences in methodology and population behaviour highlight the complexity of studying the relationship between noise exposure and health outcomes.

Regarding noise and chronic condition there is substantial evidence suggesting the link between noise exposure and cardiovascular diseases [9,20–22]. In a study published in 2023 by Stephane Buteau et al. [43], researchers attempted to investigate the potential mediation of hypertension in the relationship between noise and cardiovascular diseases. Although, they did not find a mediating effect of hypertension in the relationship, this could have been due to a lack of adjustment for lifestyle factors, such as smoking or lack of exercise. Despite this, they did find a direct effect of noise on cardiovascular diseases, such as heart attacks and strokes. Therefore, the positive association between noise and cardiovascular events aligns with our findings. In another mediation study published in 2023 by Teng Yang et al [23], they investigated the potential mediation of acute myocardial infarction in the relationship between exposure to environmental noise and the development of heart failure, they found that 12.5% of this association was mediated by having experienced a myocardial infarction in the two-year interval before. This finding supports our hypothesis, as both heart attacks and strokes can lead to the development of chronic diseases that may contribute to the onset of depression.

With respect to the relationship between chronic diseases and depression, our findings agree with the available evidence. Notice some Studies have shown a link between suffering from a chronic illness and an increased risk of depression [45–47]. In a study published in 2007 by Saba Moussavi et al [26], they found that patients suffering from one or more chronic diseases were more frequently depressed than those without a chronic illness. This was also observed in various studies [48–50]. Similarly, they found that people with chronic illness were at a higher risk of suffering from depression. The association between chronic diseases and depression could be explained by an increase in risk factors that could appear in both, for example individuals with depression might have less energy and concentration to carry out self-care activities, such as maintaining a balanced diet, exercising, and taking medication, factors that could be also found the individuals with chronic disease [51]. this also aligns with findings of higher triglyceride levels in men with depression [52] and the association of depression with hyperlipidemia [53]. Similarly, patients diagnosed with a chronic disease might experience lower mood states leading to depression upon realizing the chronicity of their illness or suffering from its complications, which could hinder their daily activities [39,40]. Finally, chronic diseases could induce depression through sleep disruption, as insomnia is a common condition in various chronic diseases [54]. The present of sleep disruption could be an important mediator in the path from chronic disease to depression and it is also related to exposure to environmental noise, however we could not include this factor in the analysis because of the high percentage of non-response in this specific question.

In the specific context of the relationship between depression and cardiovascular diseases, which are of particular interest due to their clear association with noise, various meta-analyses have demonstrated the link between depression and coronary heart disease, stroke, and cardiovascular mortality [55–57]. A study conducted in 2023 by Chayakrit Krittanawong et al. [29], found an association between depression and cardiovascular disease. Furthermore, the potential mediating role of risk factors and pathophysiological mechanisms common to both depression and cardiovascular disease, such as increased inflammatory activity, could explain this association [30].

Therefore, all the evidence discussed supports our findings and hypothesis: chronic illness, particularly chronic cardiovascular disease, may lead to depression through pathophysiological mechanisms such as increased inflammatory activity, as well as psychosocial factors associated with the illness itself.

In studies conducted on animals using very high noise levels (≥100 dB), chronic noise was shown to increase blood pressure in monkeys [58] and rats [59]. Animals exposed to noise also exhibited elevated circulating levels of angiotensin II [37,60]. Activation of the sympathetic nervous system in animals through oxidative stress generates the activation of the renin-angiotensin-aldosterone system and the subsequent release of catecholamines and hypertension [61,62]. Additionally, aircraft noise increases the expression of endothelin-1 in vascular tissue, a potent vasoconstrictor and activator of NOX-2 activity [37,63], which partly depends on the renin-angiotensin-aldosterone system[64]. Vascular and cerebral damage induced by aircraft noise was almost completely avoided by NOX2 deficiency[63] and activation of heme oxygenase-1 [65], highlighting the crucial role of inflammatory cells and oxidative stress in mediating the cardiovascular and cerebral side effects induced by noise.

In humans, the pathophysiological pathways influenced by noise are numerous, with oxidative stress and inflammation as key elements in the pathophysiology of noise due to chronic activation of stress pathways and disruption of circadian rhythms n humans, the association between noise and major cardiovascular events has been described to be mediated by the activation of amygdalar activity (part of the limbic system, involved in stress perception and emotion control), leading to vascular inflammation and severe adverse cardiovascular events [66]. In a subsequent study, the authors were able to demonstrate that greater stress resilience is associated with lower amygdala activation, vascular inflammation, and fewer severe cardiovascular events [67].

We have sought to introduce a map displaying the vulnerability levels of the districts in Madrid (Fig 2) to consider how the social determinants of health, defined by the WHO as the conditions in which people are born, grow, work, live, and age, and which influence people’s health [68], impact health inequalities. This is particularly pressing among the most vulnerable groups [69], who, as we can see on the map (Fig 2), suffer the most from the consequences of noise.

This study that we have conducted is not without limitations, some already mentioned like the not inclusion of sleep disruption data. Other important one is that the data were self-reported, this entails a memory bias when it comes to recalling information on the part of the user and possible misunderstandings or lack of comprehension of the question, which leads to the data being less reliable [70]. In contrast, as our study variable was depression, and considering the personal values in this disease, it was very important to know the user’s opinion and to find out from the user whether they had a diagnosis or not. Furthermore, in relation to noise exposure, knowing the user’s own opinion is important, as noise does not affect us all equally, and there are difficult-to-control factors such as the degree of isolation of houses or nearby noise sources. So, by asking the users opinions if they are exposed to environmental noise, we have indirectly considered these biases. Although we have not been able to study noise levels and see if there was a noise threshold beyond which people’s health was more affected. The fact that the data do not include temporal trends could be a main limitation because we do not know what came first, whether depression, chronic illness, or exposure to environmental noise. Nevertheless, there are many studies that found evidence in the proposed direction environmental noise to chronic disease and chronic disease to depression, as discussed above. But also, the fact that the time since diagnosis of a chronic condition is unknown should be considered as a limitation. Conversely, the strengths of this study include that the data obtained from the Madrid city health survey are representative of the population of Madrid, Spain, due to the use of a complex and stratified random sampling method. Additionally, the sample size is very large, with N = 8445. Lastly, the survey contains questions about many risk factors, so we have been able to control for risk factors that could be considered confounders in the relationship between noise and depression.

## Conclusion

In our study, we have found a potential link between exposure to environmental noise and depression and this relationship could be mediated by the presence of chronic disease. What our study suggests is that exposure to environmental noise would cause a chronic disease through pathophysiological mechanisms, and this in turn, through psychosocial as well as pathophysiological factors, would lead to the onset of depression. Therefore, our data suggest that it is necessary to strengthen public health interventions to reduce the population’s exposure to environmental noise and thus improve their mental health. However, prospective studies are needed to measure noise levels and include temporal data on the onset of chronic disease and depression to confirm our findings.
